# Robust Spontaneous Raman Flow Cytometry for Single‐Cell Metabolic Phenome Profiling via pDEP‐DLD‐RFC

**DOI:** 10.1002/advs.202207497

**Published:** 2023-03-04

**Authors:** Xixian Wang, Lihui Ren, Zhidian Diao, Yuehui He, Jiaping Zhang, Min Liu, Yuandong Li, Lijun Sun, Rongze Chen, Yuetong Ji, Jian Xu, Bo Ma

**Affiliations:** ^1^ Single‐Cell Center CAS Key Laboratory of Biofuels Shandong Key Laboratory of Energy Genetics Qingdao Institute of Bioenergy and Bioprocess Technology Chinese Academy of Sciences Qingdao 266101 China; ^2^ Shandong Energy Institute Qingdao 266101 China; ^3^ Qingdao New Energy Shandong Laboratory Qingdao 266101 China; ^4^ College of Life Science University of Chinese Academy of Sciences Beijing 100049 China; ^5^ College of Information Science & Engineering Ocean University of China Qingdao 266100 China; ^6^ Qingdao Single‐Cell Biotech. Co., Ltd Qingdao 266100 China

**Keywords:** high generality, high throughput, metabolic phenome profiling, Raman flow cytometry, single cell

## Abstract

A full‐spectrum spontaneous single‐cell Raman spectrum (fs‐SCRS) captures the metabolic phenome for a given cellular state of the cell in a label‐free, landscape‐like manner. Herein a positive dielectrophoresis induced deterministic lateral displacement‐based Raman flow cytometry (pDEP‐DLD‐RFC) is established. This robust flow cytometry platform utilizes a periodical positive dielectrophoresis induced deterministic lateral displacement (pDEP‐DLD) force that is exerted to focus and trap fast‐moving single cells in a wide channel, which enables efficient fs‐SCRS acquisition and extended stable running time. It automatically produces deeply sampled, heterogeneity‐resolved, and highly reproducible ramanomes for isogenic cell populations of yeast, microalgae, bacteria, and human cancers, which support biosynthetic process dissection, antimicrobial susceptibility profiling, and cell‐type classification. Moreover, when coupled with intra‐ramanome correlation analysis, it reveals state‐ and cell‐type‐specific metabolic heterogeneity and metabolite‐conversion networks. The throughput of ≈30–2700 events min^−1^ for profiling both nonresonance and resonance marker bands in a fs‐SCRS, plus the >5 h stable running time, represent the highest performance among reported spontaneous Raman flow cytometry (RFC) systems. Therefore, pDEP‐DLD‐RFC is a valuable new tool for label‐free, noninvasive, and high‐throughput profiling of single‐cell metabolic phenomes.

## Graphical abstract

Here, the development of a robust Raman flow cytometry capable of acquiring single‐cell Raman spectra (SCRS) of a variety of phenotypes rapidly is presented. By coupling with Intra‐Ramanome Correlation Analysis and deep learning, the SCRS‐based high‐throughput and high‐generality biosynthetic process dissection, antimicrobial susceptibility profiling, and cell‐type classification for isogenic cell populations of yeast, microalgae, bacteria, and human cancers are demonstrated.

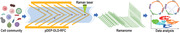

## 1 Introduction

A cell is the basic functional unit of life on Earth, thus single‐cell phenome profiling for live cell populations is of pivotal importance. We recently proposed the “ramanome” concept for metabolic phenome,^[^ ^1^ ^]^ which is a collection of single‐cell Raman spectra (SCRS; one spectrum per cell) randomly sampled from an isogenic cellular population. Each full‐spectrum spontaneous SCRS (fs‐SCRS) harbors thousands of Raman peaks, with each peak or combination of peaks potentially representing a metabolic phenotype. Therefore, a ramanome provides an information‐rich metabolic phenome for a given state of a cellular population at single‐cell resolution.^[^ ^1^, ^2^ ^]^ This phenome can include: the intake rate of hydrogen‐ and carbon‐containing substrates,^[^ ^3^ ^]^ the presence of diverse intracellular products (e.g., pigments, triglycerides, starch, and proteins),^[^ ^4^ ^]^ environmental stress responses (e.g., pathogen susceptibility to antimicrobial substances, microbial drug response mechanisms, and tumor cell drug resistance/mechanisms),^[^ ^5^ ^]^ intercellular metabolic interactions,^[^ ^6^ ^]^ etc., ramanomes can also be used to distinguish different microbial (or microalgal) species.^[^ ^7^ ^]^ Moreover, Intra‐ramanome Correlation Analysis (IRCA) can enable the construction of a metabolite interconversion network based upon subtle (yet biologically meaningful) variations among the fs‐SCRS within a single ramanome.^[^ ^8^ ^]^ Thus, ramanomes can become a universally applicable datatype of single‐cell metabolic phenome that is label‐free, noninvasive, fast, and low‐cost.

**Figure 1:**
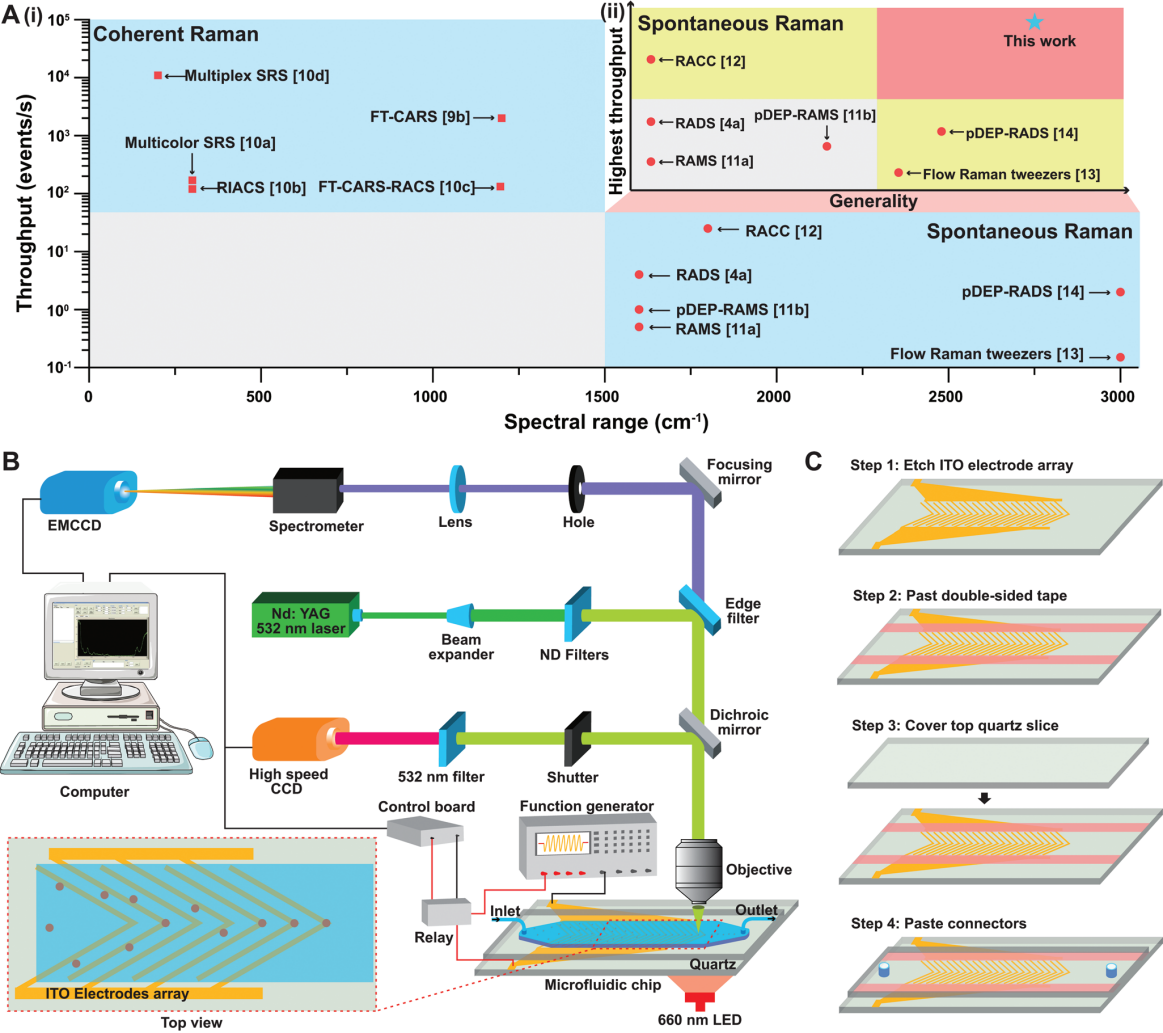
pDEP‐DLD‐RFC system setup. Ai) The throughput and spectral range of existing RFC platforms, and Aii) the throughput and general applicability of the existing spontaneous RFC platforms. However, there are few RFC platforms that are both generally applicable (able to acquire fs‐SCRS for large cell populations with long stable running time) and of high throughput. B) Schematic demonstrating the pDEP‐DLD‐RFC system setup. C) pDEP‐DLD‐RFC chip design and fabrication.

Despite these promises, acquisition of a ramanome via Raman microscopy is generally a low‐throughput method when cells are in a static mode. For example, collecting a microalgal ramanome (for pigmentation) can take 4 h to achieve a sampling depth of ≈3300 fs‐SCRS, with an acquisition time of 0.5 s per cell.^[^ ^7b^ ^]^ In contrast, Raman‐based flow cytometry (RFC) is associated with much higher ramanome acquisition throughput, e.g., at 2000 events s^−1^ for pigment spectra via coherent Raman.^[^ ^9^ ^]^ However, the use of coherent RFC is inherently limited by its narrow spectral range and low spectral resolution (i.e., poor information content and low sensitivity).^[^ ^9^, ^10^ ^]^ Spontaneous‐RFC would be an ideal solution to this problem, since it produces high throughput fs‐SCRS that cover both resonance and nonresonance Raman peaks for each of the fast‐moving cells (**Figure** **1**A).^[^ ^1^ ^]^ Unfortunately, existing spontaneous‐RFC technologies, such as Raman‐activated microfluidics sorting (RAMS),^[^ ^11^ ^]^ Raman‐activated droplet sorting (RADS)^[^ ^4a^ ^]^ and Raman‐activated cell counting (RACC), ^[^ ^12^ ^]^ are limited to only resonance Raman peaks due to use of a trap‐free approach or polydimethylsiloxane (PDMS) as structural material (which is associated with a high Raman background signal). Notably, optical tweezer‐based traps (Raman tweezers) are capable of probing mouse colon microbiota vitality (via the CD band),^[^ ^13^ ^]^ yet the throughput is limited to 3.3–8.3 cells min^−1^, due to the inability of optical tweezers (whose force is weak) to efficiently trap fast‐moving cells.

We recently reported a positive dielectrophoresis (pDEP)‐based RADS (pDEP‐RADS) system to elevate fs‐SCRS acquisition throughput.^[^ ^14^ ^]^ This system enabled yeast cell sorting via the use of quartz as structural material (to reduce Raman background), and the pDEP‐based single‐cell trap‐and‐release strategy enabled nonresonance‐band based phenotype detection (triacylglycerol (TAG) content and degree of unsaturation for fatty acids (FA‐DU)) at a throughput of ≈120 cells min^−1^. However, this system was limited by a relatively short stable running time (≈30 min) and thus unable to tackle high phenotypic heterogeneity in large cellular populations.

Toward a robust RFC system for fs‐SCRS with high accuracy, high throughput, and stable operation, here we introduce “pDEP induced deterministic lateral displacement‐based Raman flow cytometry” (pDEP‐DLD‐RFC). Tests on polymethylmethacrylate (PMMA) and polystyrene (PS) beads demonstrate excellent chemical specificity and discrimination accuracy (99.9%). Profiling of astaxanthin (AXT) content for *Haematococcus pluvialis* (*H. pluvialis*) demonstrates speed of ≈2700 events min^−1^. For isogenic populations of yeast, microalgae, bacteria (e.g., *Escherichia coli* (*E. coli*)), and human cancer cells, it produces deep, highly producible ramanomes that reveal rich, single‐cell‐resolution phenomes with high throughput. Moreover, IRCA unravels metabolic heterogeneity and metabolite‐conversion networks that are state‐ and cell‐type‐specific. The label‐free, noninvasive, information‐rich, universally applicable, and high‐throughput nature of pDEP‐DLD‐RFC promises its broad utility in metabolic profiling of cellular systems.

## 2 Results

### 2.1 The Overall Design of the pDEP‐DLD‐RFC System

A high‐robustness RFC system would require first, the operating stability with sustained running time (i.e., hours), for deep sampling of a metabolically heterogeneous cellular populations, and second, the precision of trapping fast‐moving cells of various sizes (i.e., most prokaryotic, and eukaryotic cells), and aligning them to the laser spot for efficient acquisition of a fs‐SCRS. To achieve these goals, we designed a pDEP‐DLD‐RFC system that consists of i) a Raman microscope for acquiring the SCRS of loaded cells, ii) a microfluidic chip equipped with pumps for controlling buffer flow, a function generator to produce the positive dielectrophoresis induced deterministic lateral displacement (pDEP‐DLD) force (that traps and focuses single‐cells) and a relay to control the pDEP‐DLD duration (Figure 1B).

Thus, a full pDEP‐DLD‐RFC procedure includes cell loading, pDEP‐DLD‐based cell trapping and focusing, and SCRS acquisition. The key challenge is the precipitation of cells during the procedure. In a typical microfluidic chip, the surface area of the loading pipe (hundreds of µm in diameter) can be >10 times higher than that of the channel (dozens of µm in width and in height),^[^ ^4^, ^11^, ^12^, ^14^ ^]^ leading to >10‐fold reduction of flow velocity in the loading pipe than in the channel. This results in the precipitation of cells in the loading pipe at a flow velocity that matches the pDEP‐based trapping (3–4 mm s^−1^ in the channel under the µL‐per‐hour flow rate), which is one of the key factors that contribute to low system stability (i.e., <30 min of running time).^[^ ^14^ ^]^ To overcome this problem, the pDEP‐DLD‐RFC chip was designed to contain an enlarged channel (≈1 mm in width and 50 µm in height), which enables cell loading under a high flow rate (40 µL min^−1^, under which cells can flow through the loading pipe without precipitating) whilst maintaining matchable velocity for pDEP‐based trapping. This design extends the stable running time (up to 5 h) by a up to 10‐fold. Additionally, the pDEP‐DLD‐RFC chip should allow acquiring SCRS of sufficient quality (e.g., for distinguishing nonresonance Raman peaks) yet without sacrificing throughput. To achieve this goal, fast‐moving single cells need to be trapped at the laser spot for an appropriate Raman exposure duration. Thus, we employed pDEP for the on‐and‐off trapping,^[^ ^11^, ^14^ ^]^ which has a stronger force than optical tweezers.^[^ ^13^ ^]^ Notably, the pDEP‐DLD‐RFC chip was fabricated by bonding two quartz slices with double‐sided tape, which simplified its fabrication and contributed toward higher quality SCRS by reducing the potential for Raman background signal (Figure 1C; and Figure S1, Supporting Information).

Furthermore, the chip should support high coverage of cells for SCRS acquisition, i.e., ensuring none or few cells miss or skip Raman detection. Specifically, the flowing cells should be precisely focused to the laser spot, which can be challenging to obtain in a wide channel. Among the potential solutions, hydrodynamic focusing usually results in very high cell velocity which would reduce the cell trapping efficiency.^[^ ^4^, ^11^, ^14^ ^]^ Acoustofluidic focusing can focus cells at a velocity that is suitable for trapping; however, this type of system cannot be easily integrated into our pDEP‐based chip due to the complexity required for fabricating and reconciling acoustic electrodes and pDEP electrodes simultaneously.^[^ ^9^, ^10^ ^]^ Therefore, we adopted a pDEP‐DLD strategy,^[^ ^15^ ^]^ in which sloped electrodes were incorporated in our pDEP chip to deflect the flowing cells and focus them to the laser spot in a strict sequential manner. Specifically, an electrode array (rather than a single electrode pair) was designed to be symmetrically slopped and intersect at a 30° angle (Figure 1B). Indium tin oxide (ITO) was selected to form the electrodes, which is more conducive to high‐quality SCRS acquisition than metals, such as gold, silver, and copper, which can cause photothermal damage to cell vitality under the 532 nm laser.^[^ ^14^ ^]^ With these designs, the efficiency of trapping and focusing is >90% under an alternating current (ac) of 18 Vp‐p (voltage peak‐peak) at 1.0 MHz (Movie S1, Supporting Information), setting the stage for efficient SCRS acquisition. Finally, the SCRS acquisition time and the trapping duration within the pDEP‐DLD‐RFC need to be precisely aligned to ensure that signal detection and cell movement is fully synchronized during high‐speed flow. For this, a continuous pDEP (as opposed to periodical) was exerted to trap a cell and then release it by periodically triggering relay off based on the acquisition time (Figure S2 and Movie S2, Supporting Information). Therefore, over our previously reported pDEP‐RADS system,^[^ ^14^ ^]^ the pDEP‐DLD‐RFC has several significant advances: i) the integration of wide (≈1 mm in width) loading channel with pDEP‐DLD focusing has improved the stable running time greatly (from 30 min to 5 h), yet maintained high detection efficiency (>90%), avoided clogging by debris and consequentially facilitates chip reuse (to >20 times); ii) the coupling of continuous pDEP‐based single‐cell trap and acquisition time‐dependent release, rather than the functional generator self‐generated periodical pDEP‐based trap‐and‐release (where the trapping duration has to be set as double the acquisition time, to ensure each cell undergoing a complete Raman exposure period), has increased the throughput and the accuracy; iii) the adoption of double‐sided tape has simplified chip fabrication and reduced the cost. These aforementioned factors form the basis of the pDEP‐DLD‐RFC, which we hypothesized (and subsequently demonstrated herein) would provide a more robust, operationally stable RFC system for accurate and high‐throughput fs‐SCRS acquisition.

### 2.2 Benchmarking the pDEP‐DLD‐RFC Platform

**Figure 2:**
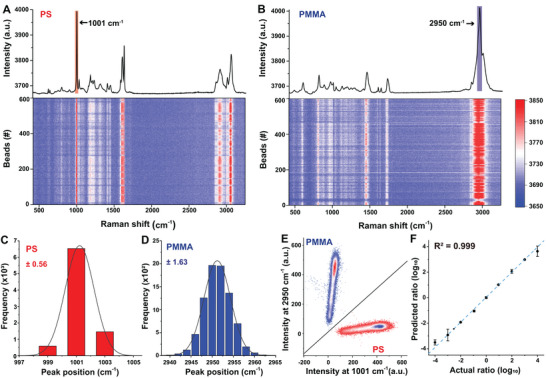
pDEP‐DLD‐RFC accuracy evaluation using beads. The average spectrum (top, *n* >8000) and 600 randomly selected single bead spectra (bottom) for A) PS and B) PMMA beads. C) The standard deviation around the 1001 cm^−1^ peak position was 0.56 for the PS beads and D) 1.63 for PMMA beads around the 2950 cm^−1^ peak. E) PS (*n* >8000) and PMMA (*n* >8000) beads intensities at 1001 and 2950 cm^−1^, demonstrating a high discrimination accuracy of 99.9%. F) Liner correlation between the actual and Raman predicted ratio for the PS : PMMA (from 1:10 000 to 10 000:1) beads, *R* ^2^ = 0.999. Error bars indicate the SD of three independent experiments.

PS and PMMA beads were used to benchmark pDEP‐DLD‐RFC performance, which have been widely used for evaluating RFC/RACS platforms.^[^ ^9^, ^10^ ^]^ First, the PS and PMMA beads were loaded (separately) and characterized at a throughput of ≈2700 events min^−1^ (2 ms acquisition time plus 20.2 ms for data recording and saving; *n* > 8000). Under these conditions, the signal‐to‐noise ratio (SNR) averaged >6.1‐ and >6.7‐fold (signal : background) for the PS and PMMA beads, respectively, which is indicative of a high‐quality Raman signal. The characteristic 1001 cm^−1^ peaks (assigned as phenyl ring breathing mode) for the PS beads (**Figure** **2**A), and 2950 cm^−1^ (assign as C—H stretch) for the PMMA beads (Figure 2B), were detected with only minor inter‐bead Raman spectra fluctuations. The standard deviation at peak positions 1001 cm^−1^ (PS, Figure 2C) and 2950 cm^−1^ (PMMA, Figure 2D) were just 0.56 and 1.63 cm^−1^, respectively. In fact, these intensity readings enable the classification of PS beads and PMMA beads with an accuracy of >99.9%, only three of the >16 000 beads were misclassified (Figure 2E). These results indicate that the pDEP‐DLD‐RFC is stable and capable of acquiring highly specific and reproducible SCRS.

The sensitivity of pDEP‐DLD‐RFC was tested using a PS and PMMA bead mixture (with the *n* in each measurement ranging from >400 for 1:1 mixtures to >30 000 for 1:10 000 and 10 000:1 mixtures). The type of each bead (PS or PMMA) within the mixtures was predicted individually based upon an analysis of the intensity reading. Classification of the mixture by subtracting the *I*_2950_ readings from those of the *I*_1001_ (thus, PS were identified by readings of >0, and PMMA by those <0) demonstrated a liner relationship (*R* ^2^ = 0.999) between the actual and the predicted ratios (Figure 2F). Thus, the pDEP‐DLD‐RFC enabled an accurate and sensitive detection of mixed sample composition, albeit one with a highly biased population structure.

**Figure 3:**
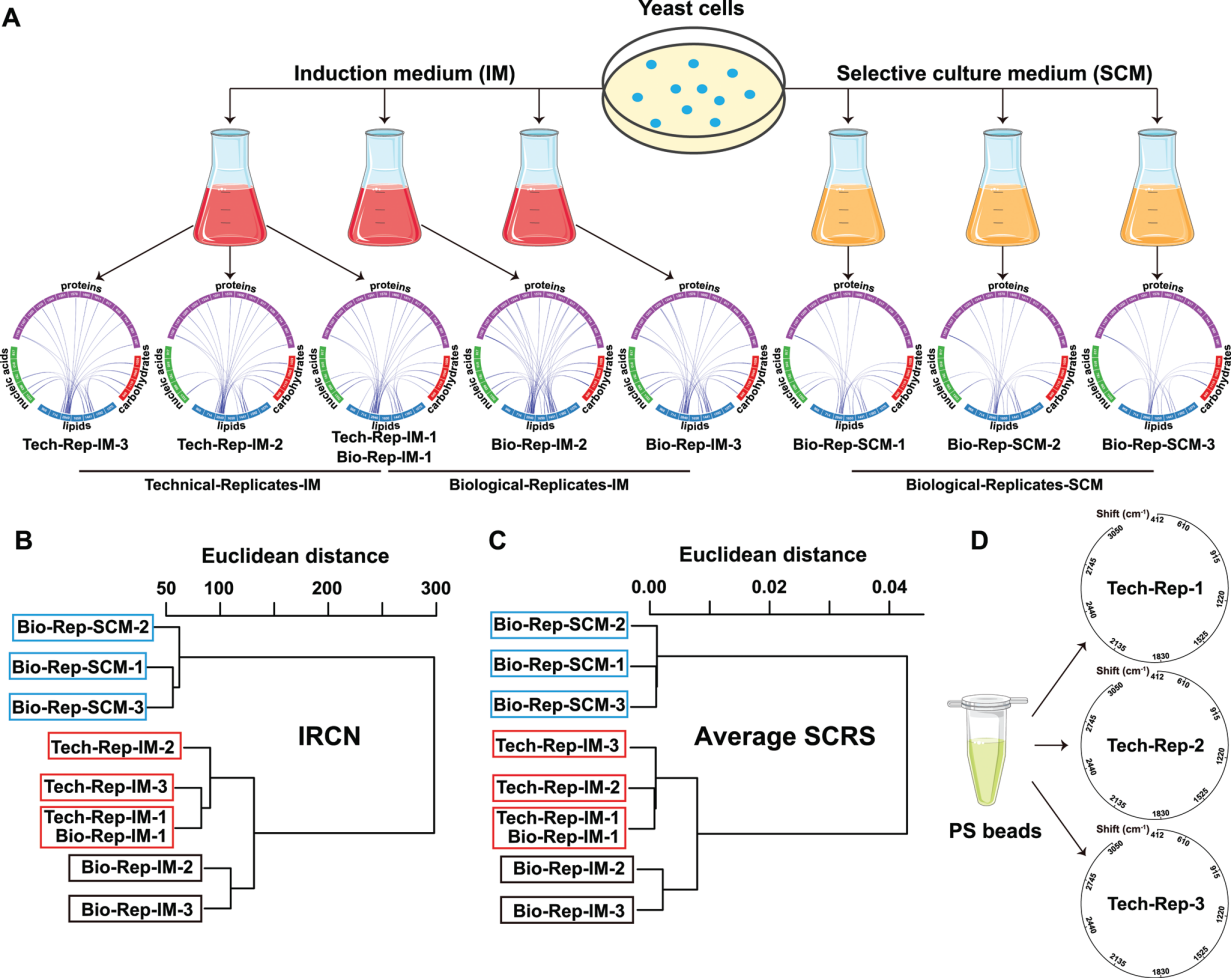
pDEP‐DLD‐RFC reproducibility evaluations. A) The IRCA for *NoDGAT2A*‐expressing yeast cells were generated using three technical replicates in IM, three biological replicates in IM and three biological replicates in SCM. Clustering patterns via HCA are shown based upon the B) IRCN correlation matrix or C) average SCRS. D) Three technical PS bead IRCA replicates.

Transgenic oleaginous *Saccharomyces cerevisiae* that expresses *NoDGAT2A* (an oil‐producing diacylglycerol acyltransferase found in the industrial microalga, *Nannochloropsis oceanica*) were used to benchmark cellular metabolic profiling.^[^ ^16^ ^]^ The schematic in **Figure** **3**A demonstrates the yeast culture strategy using Induction Medium (IM; in which TAG synthesis is induced) or Selective‐culture Medium (SCM; as a negative control). The fs‐SCRS were acquired (acquisition time of 200 ms plus 20.2 ms for data recording and saving) for each culture condition at a throughput of ≈270 cells min^−1^ (*n* >1000 for each of three technical and three biological replicates per culture condition). A high signal quality was observed during the ramanome acquisition, with an average SNR of >4.2‐fold. The within‐group variation (quantified via Euclidean distance) was significantly lower (*p* ≤ 0.05) than the between‐group variation for both the technical replicates and biological replicates (Figure 3B,C). Furthermore, the variation among the biological (and technical) replicates within the IM group were lower than those between the IM and SCM (*p* ≤0.05; Figure 3B,C) and confirmed that the culture medium was responsible for differences observed via the analysis of the two groups (i.e., TAG‐producing vs non‐TAG‐producing). These results indicate that the ramanome acquisition was highly reproducible.

We have previously shown that an Intra‐ramanome Correlation Network (IRCN), derived from an IRCA, is a reliable, species‐resolved, and state‐sensitive intrinsic metabolic signature for isogenic cellular populations.^[^ ^8^ ^]^ An IRCN can reveal vast information‐rich networks by pair‐wise correlations of potential metabolite interaction peaks within fs‐SCRS that were sampled from just one instance of a cellular population.^[^ ^8^ ^]^ IRCNs were derived for each of the yeast ramanomes, and active metabolite conversions are found in all the yeast samples (Figure 3A). The IRCN conversion patterns were identical among the three tests (*n* >1000 per test), i.e., the IM technical replicates, IM biological replicates, and SCM biological replicates (Figure 3A; and Figure S3, Supporting Information), which further supported the assessment of data reproducibility. A far greater number of correlations were identified for the IM yeast in the IRCN between protein‐ and lipid‐peaks, when compared to the SCM. This observation is consistent with the condition‐specific TAG production by the *NoDGAT2A*‐overexpression yeast that is only induced by the IM.^[^ ^16^ ^]^ Moreover, clustering based upon the IRCNs (via the inter‐peak correlation matrix; Figure 3B) or the average fs‐SCRS in a ramanome (Figure 3C), readily discriminated the yeast between not only the different culture conditions, but also the intracondition technical and biological replicates. Naturally, the IRCA did not reveal any metabolite conversions for the PS beads among the three tests (*n* >1800 for each test; Figure 3D).

### 2.3 Resonance‐Raman Peak‐Based Metabolic Profiling Capabilities

Resonance Raman peaks have a much stronger signal intensity than nonresonance peaks within an fs‐SCRS, which can be used to identify the presence of compounds that generate resonance Raman bands (e.g., pigments) and frequently serve as marker bands for computing the intake‐rate of stable‐isotope labeled substrates.^[^ ^3^, ^17^ ^]^ Thus, an AXT‐producing *H. pluvialis*, a unicellular freshwater green microalga,^[^ ^18^ ^]^ was employed as a model to examine the pDEP‐DLD‐RFC's resonance Raman peak profiling capabilities. Although the AXT‐producing *H. pluvialis* can be measured in a continuous flow mode, due to its strong resonance Raman signal that requires as short as 2 ms acquisition time, a longer time of 20.2 ms is still required to record/save data, which would cause many cells to miss Raman detection. Hence, to maintain high detection efficiency, the trap‐and‐release mode was used to arrange the single cells to pass through the laser point every 22.2 ms (acquisition time of 2 ms plus 20.2 ms for data recording and saving) apart.

**Figure 4:**
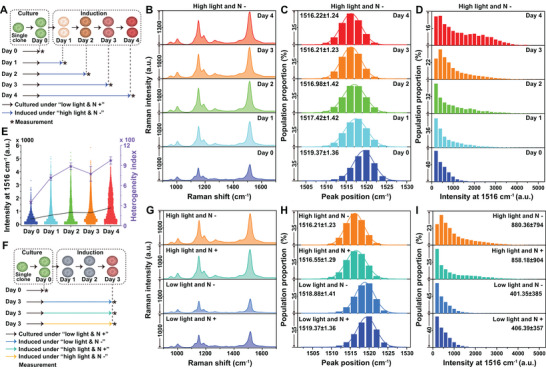
High‐throughput label‐free profiling for AXT‐producing *H. pluvialis*. A) Cell preparation procedure using various induction times with high light and nitrogen depletion. B) The average SCRS for *H. pluvialis* cells (*n* >20 000) cultured for 0 to 4 days under high light and nitrogen deficiency stress, which caused a gradual increase in AXT synthesis over time. C) Peak position distribution around 1516 cm^−1^ during the 4‐day cultivation, which demonstrated a prominent shift from 1519 to 1516 cm^−1^. D) AXT‐production by *H. pluvialis* cells during the 4‐day cultivation. E) AXT‐content heterogeneity (indicated by the intensity at 1516 cm^−1^) during the 4‐day cultivation. F) The cell preparation procedure, with a 3‐day induction under a combination of stress conditions. G) The average SCRS for *H. pluvialis* under different stress conditions for 3 days, during which high light had a larger effect on AXT production than nitrogen deficiency. H) Peak position distribution around 1516 cm^−1^ for the various stress conditions. I) AXT production by *H. pluvialis* under various stress conditions.

AXT production by *H. pluvialis* is known to be condition‐dependent, thus the microalgal cells were cultured with a combination of two environmental factors (light intensity and nitrogen level): i) low intensity lighting (low light) and nitrogen repletion (N +), ii) low light and nitrogen depletion (N −), iii) high intensity lighting (high light) and N +, and iv) high light and N − (**Figure** **4**A,F). The temporal culture samples were profiled using the pDEP‐DLD‐RFC chip, whereby >20 000 *H. pluvialis* cells were acquired within 15 min to obtain a ramanome for each sample. The AXT cellular content was measured based upon its resonance Raman signal, which includes two prominent characteristic bands at 1516 and 1157 cm^−1^ that signify C=C and C—C stretching vibrations of the chain bands, respectively.^[^ ^19^ ^]^ Among the four culture conditions tested, “high light and N−” is associated with AXT induction. Under these conditions, the averaged SCRS recorded a temporal‐based increase at 1516 and 1157 cm^−1^ (Figure 4B; and Figures S4A and S4B, Supporting Information), confirming that AXT accumulated in the cells. Tracking AXT‐peaks over time at single‐cell resolution revealed a time‐dependent shift from 1519 to 1516 cm^−1^ (Figure 4C), while the peak 1157 cm^−1^ remained unchanged (Figure S4C, Supporting Information).

Moreover, AXT‐content heterogeneity shows that cells with high‐AXT‐content were gradually enriched in the population (Figure 4D; and Figure S4D, Supporting Information), with a faster accumulation rate during days 0–1 and days 3–4 (Figure 4E), Throughout the course of the experiment a substantial number of cells remained nonproductive, with AXT‐low/negative cells (*I*_1516_ <600) representing 76.76%, 61.02%, 50.15%, 49.15%, and 35.16% from day 0 to 4, respectively. Notably, inter‐cellular AXT‐content heterogeneity also increased with its accumulation (Figure 4E).

A comparison of the ramanomes enabled the detection of differences between the four conditions (Figure 4G–I; and Figure S5, Supporting Information): i) high light was more effective than nitrogen level as a means of stimulating AXT production, with the low light conditions being ineffective (Figure 4G); ii) the time‐dependent shift from 1519 to 1516 cm^−1^ was apparent only for the high light cultures, and consistent with AXT accumulation (Figure 4H); iii) high light with nitrogen depletion still enhanced AXT production (Figure 4I). These results underscore the value of pDEP‐DLD‐RFC for quantitative and heterogeneity‐resolved monitoring of resonance‐Raman peaks.

### 2.4 Nonresonance‐Raman Peak‐Based Multiphenotype Profiling

TAG‐synthetic yeast cells were used to model the pDEP‐DLD‐RFC's performance for profiling phenotypes associated with non‐resonance Raman peaks, which often have a 10^6^‐fold weaker signal than resonance peaks. After inoculating a single colony of the *NoDGAT2A*‐expressing yeast into SCM at 30 °C and 200 rpm for 12 h, cellular TAG accumulation was induced by changing the culture medium to IM. The temporal ramanome changes for *n* >3500 *NoDGAT2A*‐expressing yeast following TAG induction was monitored using the pDEP‐DLD‐RFC chip (Figures S6 and S7, Supporting Information) at a throughput of ≈270 events min^−1^ (with an acquisition time of 200 ms per cell, plus 20.2 ms for data recording and saving).

The TAG content within each cell was measured via its characteristic peaks at 2853 cm^−1^ (assigned as C—H_2_ and C—H_3_ asymmetric and symmetric stretches) (Figure S6A, Supporting Information). The number of cells with high‐TAG‐content were gradually enriched in the population (Figure S6B, Supporting Information). However, a substantial number of cells were non‐/low‐TAG‐producing (*I*_2853_ <0) each day, with non‐/low‐TAG‐producing cells representing 99.97% of the uninduced populations and 71.75–49.92% of the induced populations. Tracking the distribution around 2853 cm^−1^ further revealed a temporal incremental increase for: i) the percentage of TAG‐synthetic (defined as *I*_2853_ – *I*_2866_ >0) cells (Figure S6C, Supporting Information), ii) average TAG content (indicated by averaged “*I*_2853_ – *I*_2866_”; Figure S6C, Supporting Information), and iii) the level of heterogeneity (Figure S6B, Supporting Information) during the 5‐day cultivation.

The degree of unsaturation (DU) for fatty acids can be derived for each cell by assessing the ratio between the peaks associated with the C=C stretch (1665 cm^−1^) and CH_2_ bend (1445 cm^−1^), i.e., “(*I*_1655_ *– I*_1800_) */* (*I*_1445_ *– I*_1800_)”,^[^ ^14^, ^20^ ^]^ This calculation demonstrated that: i) the a greater number of low‐DU‐TAGs were produced as TAG synthesis progressed (Figure S7, Supporting Information), which is consistent with NoDGAT2A preference for unsaturated‐fatty acids;^[^ ^16^ ^]^ ii) The sharpest DU decrease was from day 0 to 1 (Figure S7B, Supporting Information), during which time the fastest TAG content increases occurred (Figure S6, Supporting Information); iii) while the number of high‐TAG‐content cells were gradually enriched (Figure S6B, Supporting Information), a substantial number of cells (99.32% for uninduced populations and 78.55% to 73.54% for induced populations) had a high DU (>0.5) each day (Figure S7B, Supporting Information).

The CDR (CD ratio) within a fs‐SCRS can be tracked within a ramanome following cellular exposure to heavy water (D_2_O). The CDR is defined as the ratio between the CD band area (2040–2300 cm^−1^) and the CD plus CH bands (2800–3050 cm^−1^) area.^[^ ^3^, ^5^, ^13^, ^21^ ^]^ A longer acquisition time of 2 s per cell (throughput of ≈30 events min^−1^) was required to profile the yeast cells after D_2_O feeding because the Raman signal within the CD band area is generally weaker (≈3.5‐fold lower) than typical spontaneous peaks (e.g., the TAG peak at 2853 cm^−1^, C=C stretch at 1665 cm^−1^ and CH_2_ bend at 1445 cm^−1^). A high‐quality fs‐SCRS (average SNR of >3.0) could still be obtained with the extended acquisition time. The CDR distribution pattern was consistent with the procession of growth phases through a typical growth curve, thus i) the CDR was higher than the control group for the D_2_O supplemented cells (indicating D_2_O intake) at both the 12 and 36 h time points; ii) the CDR was higher at the 12 h time point than that of the cells at the 36 h time point, suggesting a higher metabolic activity; iii) the cellular heterogeneity was high at 36 h than at the 12 h timepoint (Figure S8, Supporting Information).

**Figure 5:**
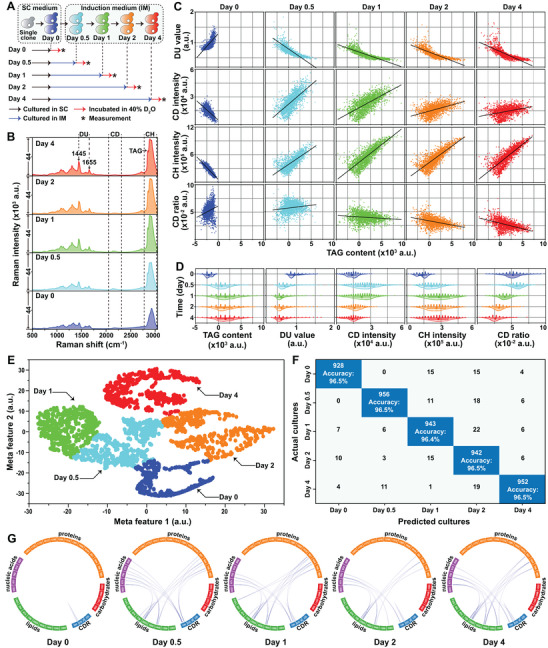
Metabolic phenome profiling (TAG productivity, DU value, and metabolic activity) for oleaginous yeast. A) TAG‐producing yeast cells were prepared using different induction periods and incubated with D_2_O under identical conditions. B) The average SCRS for D_2_O labeled TAG‐producing yeast cells (*n* >1800) show a 4‐day a dynamic change in multiphenotypic heterogeneity. C) Scatterplots and liner analysis of TAG content (represented by “*I*_2853_ *– I*_2866_”), DU value (represented by “(*I*_1655_ *– I*_1800_) */* (*I*_1445_ *– I*_1800_)”), and metabolic activity (represented by CD intensity, CH intensity, and CD ratio). D) Distribution of TAG content, DU value and metabolic activity. E) The *t*‐SNE plots after applying CNN on the SCRSs (≈900 per culture for training; ≈900 per culture for analysis). F) Confusion matrix for the cultures. The CNN identified and classified the different cultures with a classification accuracy of >96.4%. G) The local IRCNs for yeast cells following various induction durations.

The capabilities of the pDEP‐DLD‐RFC to generate rich and comprehensive fs‐SCRSs for simultaneous profiling of multiple phenotypes was explored (at a throughput of ≈30 events min^−1^) in *NoDGAT2A*‐expressing yeast cells that were both TAG induced and supplemented with 40% D_2_O (**Figure** **5**A). Over 1800 fs‐SCRS per condition were used for the phenome (TAG content, DU value, CD intensity, CH intensity, and CDR) correlation analysis (Figure 5B–D). Increased TAG‐content within a cell was correlated with: i) decreased DU value, indicating the presence of low‐DU TAG; ii) increased CD intensity, indicating faster D_2_O intake for cells with higher TAG‐synthesis activity; iii) increased CH intensity, indicating a total lipid increase due to TAG accumulation; iv) A temporal CDR increase (prior to day 0.5) and subsequent decrease (after day 1), indicating a positive correlation between TAG‐synthesis and metabolic activity at an early stage and negative correlation at later stages (due to the reduced cellular vitality following excessive TAG accumulation). While there was large cell‐to‐cell phenotype heterogeneity for each of the samples (Figure 5C,D), the heterogeneity of all measured phenotypes (TAG content, DU value, and metabolic activity) increased tremendously during the first 12 h. Two distinct temporal patterns were particularly notable: i) cells with a TAG content, DU value, CH intensity, and CDR that remained largely unchanged, and ii) those with a slight CD intensity decrease (Figure 5C,D).

A six‐layer convolutional neural network (CNN) structure (with fs‐SCRS feature compression and extraction) was used to test whether the cultures (and cellular states) can be distinguished via a ramanome analysis. The scatterplots generated for the extracted features were assessed through *t*‐distributed stochastic neighbor embedding (*t*‐SNE), which indicated a clear separation between the different cultures (Figure 5E). Notably, an accuracy of >96.4% was achieved when identifying the cultures (Figure 5F). This analysis revealed that the pDEP‐DLD‐RFC can support multiphenotype monitoring to revealing dynamic interactions, including metabolite conversions and metabolic state‐based classification of cellular systems.

The IRCNs for these samples revealed characteristic metabolic conversions at each time point. The cells at day 0 were metabolically inert, with few conversions; yet many conversions were observed as early as 12 h. There were numerous conversions among lipids, proteins, nucleic acids, and CDR, with lipids serving as a crucial node that connects to many other compounds. After the first day, the conversions were reduced, particularly among those involving nucleic acids. The conversions were further reduced after the second day, especially among those involving proteins (Figure 5G; and Figure S9, Supporting Information).

### 2.5 Nonresonance‐Raman Peak‐Based Profiling in Bacteria

*E. coli* supplemented with 50% D_2_O and 32 mg L^−1^ ampicillin (Amp), 16 mg L^−1^ gentamicin (Gen), or 2 mg L^−1^ levofloxacin (Lev) were used to assess the fs‐SCRS acquisition capabilities when small cells were analyzed with the pDEP‐DLD‐RFC. A 2 s acquisition time was used to analyze >1200 cells per sample, which represented a throughput of ≈30 events min^−1^. The average SNR was > 3.5 for the bacteria, indicating high‐quality data.

**Figure 6:**
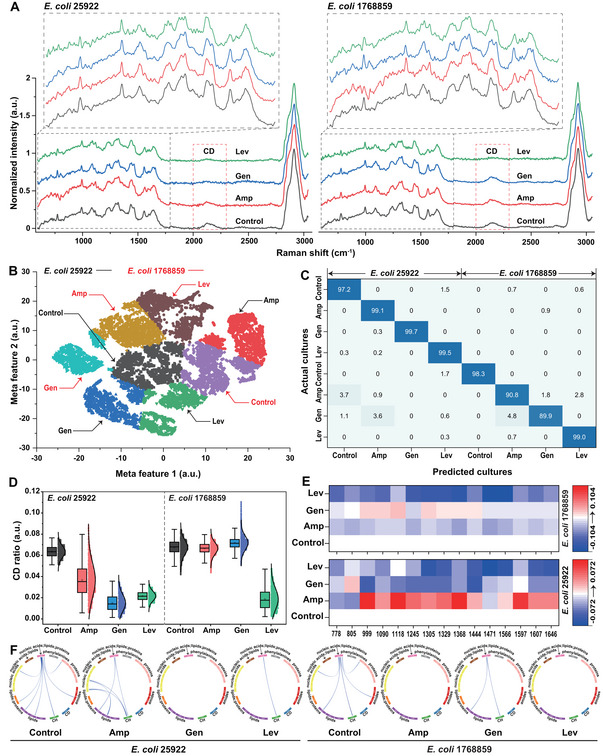
*E. coli* antimicrobial susceptibility tests. A) The average SCRS for the two *E. coli* strains following treatment with different drugs (*n* >1200). B) A *t*‐SNE plot for the cells following the application of the CNN to the acquired SCRSs (≈600 per culture for training; ≈600 per culture for analysis). C) Confusion matrix for the cultures. The CNN identified and classified the different culture types with a classification accuracy of 89.9–99.7%. The D) CDR, E) RBCS, and F) local IRCNs for the different cultures following incubation with the different drugs.

The fs‐SCRS captured the characteristic of *E. coli* “fingerprint” region between 550 and 1800 cm^−1^, plus the CD band region (2040–2300 cm^−1^) that can indicate drug susceptibility (**Figure** **6**A). The fs‐SCRS scatterplot (generated by a *t*‐SNE to extract features from across the full‐spectrum) indicates a clear separation between the cultures (Figure 6B), with a discriminating accuracy of 89.9–99.7% and average of 96.69% (Figure 6C). Thus, the pDEP‐DLD‐RFC was capable of distinguishing bacterial species‐ and state‐specific features (i.e., drug‐responses).

A heterogeneity‐resolved CDR analysis revealed decreased metabolic activity was observed for all drug‐treated groups versus the control group for the ATCC 25 922 *E. coli*, whereas only the Lev‐treated group was decreased for the 1 768 859 *E. coli* (Figure 6D). The signatures were consistent with existing antimicrobial susceptibility test (AST) reports for the broth microdilution approach, i.e., *E. coli* ATCC 25 922 sensitivity to Amp (32 mg L^−1^), Gen (16 mg L^−1^), and Lev (2 mg L^−1^), and *E. coli* 1 768 859 resistance to Amp (32 mg L^−1^) and Gen (16 mg L^−1^) and sensitivity to Lev (2 mg L^−1^). Furthermore, the Raman barcode of cellular‐response to stress (RBCS)‐based analysis illustrated that the different drugs induced distinct cellular responses (Figure 6E). Comparison of RBCS revealed that Amp (32 mg L^−1^) induced the largest number of Raman peak increases (e.g., 990 (C—C stretching), 1090 (C—C stretching), 1118 (C—C stretching), 1245 (Amide III), 1305 (CH_3_ and CH_2_ twisting), 1329 (CH_3_CH_2_ wagging mode), 1368 (CH_3_ stretching), 1444 (CH2 bending), 1597 (Amide I), 1607 (C=C stretching), 1646 (Amide I) cm^−1^); whereas Gen (16 mg L^−1^) induced the smallest number of Raman peaks increases (e.g., at 805 (backbone geometry and phosphate ion interactions) cm^−1^) (Figure 6E; bottom). However, this pattern was not observed in the Amp and Gen resistant *E. coli* 1 768 859 (Figure 6E; top). On the other hand, Lev (2 mg L^−1^) induced a Raman peak decrease in both *E. coli* ATCC 25 922 and *E. coli* 1 768 859 (Figure 6E).

An IRCA for these samples revealed distinct metabolite‐conversion networks among the cultures (Figure 6F). All the drugs impacted the metabolite‐conversions to a certain degree. Specifically, Amp (32 mg L^−^) triggered conversions between nucleic acids and lipids in the Amp‐sensitive *E. coli* strain (ATCC 25 922). This analysis reveals that the pDEP‐DLD‐RFC can facilitate high‐throughput identification and classifications of drug‐response mechanism.

### 2.6 Label‐Free Noninvasive Characterization and Discrimination of Single Cancer Cells

To test the feasibility of fs‐SCRS based cancer cell discrimination with the pDEP‐DLD‐RFC chip, human bladder (T24), lung (A549), renal (OSRC‐2), and breast (MCF‐7) cancer cell lines were assessed following culture under identical conditions. A 2 s acquisition time (≈30 events min^−1^) was applied to analyze >1800 cells for each cell line. High quality data were obtained, with an average SNR of >3.1.

**Figure 7:**
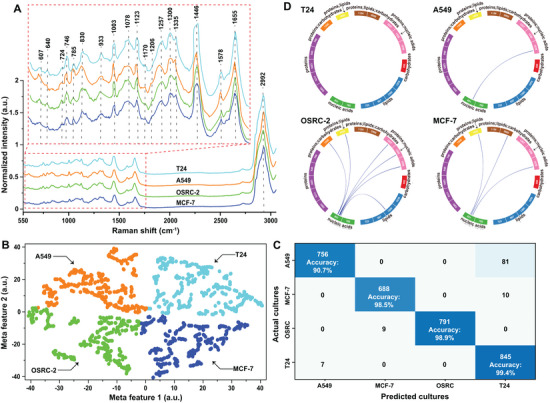
Cancer cell line profiling. A) The average SCRSs for the T24, A549, OSRC‐2, and MCF‐7 cells (*n* >1800). B) The *t*‐SNE plot for the cells after applying a CNN on the acquired SCRSs (≈900 per culture for training; ≈900 per culture for analysis). C) Confusion matrix for the cultures. The CNN identified and classified the cancer cell lines with a classification accuracy of 90.7–98.9%. D) The IRCNs for the various cancer cell lines.

The fs‐SCRS acquired during this analysis revealed details within the “fingerprint” region (550–1800 cm^−1^) that were characteristic for each of the T24, A549, OSRC‐2, and MCF‐7 cell lines (**Figure** **7**A). The fs‐SCRS scatterplots generated from the features extracted by the *t*‐SNE analysis, indicated a clear separation between the cultures (Figure 7B), with an accuracy of 90.7–99.4% and average of 96.9% (Figure 7C).

The IRCA analysis for these cancer ramanomes revealed distinct metabolite‐conversion networks (Figure 7D; and Figure S10, Supporting Information). Specifically, the IRCNs (Figure 7D) revealed that: i) no conversions were observed for T24; ii) only conversions from nucleic acids to proteins were found for A549; iii) relatively more conversions are present for OSRC‐2, including those from nucleic acids to proteins, carbohydrates, and lipids; iv) relatively fewer conversions were present for MCF‐7, involving mainly those from nucleic acids to proteins, carbohydrates, and lipids. Therefore, the pDEP‐DLD‐RFC can distinguish different types of cancer cells, unveil metabolic heterogeneity between cancer cell populations, and provide mechanistic insights in a label‐free and high‐throughput manner. As a fs‐SCRS is sensitive to the metabolic state of a cell, it would be of interest to test whether pDEP‐DLD‐RFC can distinguish among the subtypes of a tumor, or identify cells in a particular developmental state (such as those undergoing epidermal mesenchymal transition) in the future.

## 3 Discussion

Ramanomes represent a new single‐cell big‐data type for culture‐independent, label‐free, noninvasive, and information‐rich phenomes that can be applied to all cell types.^[^ ^1^, ^2^ ^]^ RFC is a promising tool for this purpose,^[^ ^9a^ ^]^ yet existing RFC platforms are either of low throughput or unable to acquire fs‐SCRS from large cell populations. This article introduced a pDEP‐DLD‐RFC system that overcomes these limitations by designing a chip with an enlarged channel size (≈1 mm in width and 50 µm in height) to load cells at a high flow rate (40 µL min^−1^), avoiding cell precipitation in the loading pipe, enabling an extended stable running time, while maintaining a slow velocity that is conducive to the efficient trapping of fast‐moving single cell. The exertion of a pDEP‐DLD force (rather than just pDEP force^[^ ^14^ ^]^) via a sloped electrode array enabled the trapping, focusing, and arrangement of fast‐moving single cells for efficient fs‐SCRS acquisition. Moreover, the use of quartz as the primary structural material enabled the pDEP‐DLD‐RFC to further increase phenotype profiling accuracy by reducing Raman background signals. The long stable running time (up to 5 h) associated with this platform enables the detection of rare target cells in a large population and promotes the commercial viability of the system. The pDEP‐DLD‐RFC design extends the stable running time by a up to 10‐fold compared with the pDEP‐RADS system (≈30 min).^[^ ^14^ ^]^

Many ramanome analyses are dependent upon a sufficient sampling depth, including IRCA, metabolite‐content quantification, and heterogeneity index computation.^[^ ^22^ ^]^ Moreover, a high sampling depth is essential for applications requiring high sensitivity, such as those in which rare target cells (with distinct fs‐SCRS) need to be identified from a large population. The pDEP‐DLD‐RFC chip was demonstrated to readily achieve a fs‐SCRS sampling depth composed of thousands of cells. Therefore, it is possible to use this system to capture dynamic metabolic features rapidly, accurately, and comprehensively from just one instance of an isogenic cellular population. The pDEP‐DLD‐RFC can process and enable cancer cell lines to be distinguished at a higher throughput than the rate described for spontaneous RFC (i.e., ≈30 events min^−1^ vs <6 cells min^−1^, respectively).^[^ ^23^ ^]^ Additionally, we demonstrated that the AXT‐content of *H. pluvialis* cells can be profiled via resonance Raman peaks at ≈260 cells min^−1^ using RADS.^[^ ^4a^ ^]^ In comparison, the pDEP‐DLD‐RFC further elevated the throughput to ≈2700 events min^−1^ (a >10‐fold increase), where resonance Raman spectra were acquired and recorded every 22.2 ms (including 2 ms for acquisition plus 20.2 ms for data recording/saving). Notably, the 10‐fold higher throughput did not compromise the quality of resonance spectrum, which was produced with an average SNR of >4.5 folds and is indicative of a high quality SCRS signal.

Furthermore, the pDEP‐DLD‐RFC is capable of overcoming the major challenges that bacterial cells can pose to RFC based systems. The focusing and accurate alignment of individual bacterial cells to the laser is difficult due to their small size (≈1 µm).^[^ ^10b^ ^]^ The CD band and the “fingerprint” region have been used for AST and for revealing drug response mechanisms via RBCS, respectively, in bacteria.^[^ ^5^, ^24^ ^]^ The results produced via pDEP‐DLD‐RFC here are consistent with our past observations of cellular response after Amp treatment (i.e., the sharp decrease in intensity of nucleic acid‐related bands, while no change or an increase of lipid‐related bands, which is perhaps linked to the Amp associated inhibition of cell wall/membrane synthesis).^[^ ^5^, ^25^ ^]^ Previously, the acquisition of fs‐SCRS from drug‐exposed or D_2_O‐fed bacterial cells was typically conducted at a low throughput (≈3–5 cells min^−1^) using static cells on a substrate (e.g., CaF_2_).^[^ ^5^, ^24^ ^]^ An automated flow‐based Raman platform reported a similarly low throughput of 3.3–8.3 cells min^−1^, due to the inability of optical tweezers to efficiently trap fast‐moving cells.^[^ ^13^ ^]^ Notably, the pDEP‐DLD‐RFC greatly improved this rate to ≈30 events min^−1^, representing a significant elevation in throughput.

It is notable that our pDEP‐DLD‐RFC system is still limited in its ability to tackle those very small cells (i.e., ≤1 µm), whose throughput remained relatively low as compared to the larger cells (e.g., the ≈2700 events min^−1^ achieved for *H. pluvialis*) that were analyzed with this system. This was especially true for profiling nonresonance‐Raman peak‐based phenotype due to the long acquisition time demanded for obtaining high‐quality fs‐SCRS. Therefore, further development will focus upon: i) either coupling pDEP‐DLD with acoustic focusing^[^ ^9^, ^10^ ^]^ or 3D focusing^[^ ^13^ ^]^ to align the small cells more efficiently; ii) integrating pDEP‐DLD with surface‐enhanced Raman scattering (SERS) to greatly reduce the acquisition time for each cell; iii) incorporating a line‐focusing strategy to detect multiple cells for SCRS in parallel,^[^ ^26^ ^]^ and iv) either coupling to pDEP‐RADS^[^ ^14^ ^]^ to support sustained high‐throughput Raman‐activated cell sorting (RACS), or to a droplet‐based dispenser^[^ ^27^ ^]^ for on‐demand, indexed “one‐cell‐one‐tube” exportation of target‐cells for downstream single‐cell sequencing or cultivation.^[^ ^21^ ^]^ Finally, v) the fs‐SCRS can be integrated with impedance or mass to achieve high‐speed multimodal cellular profiling.^[^ ^28^ ^]^

Nevertheless, we showed that the pDEP‐DLD‐RFC chip can support a wide variety of applications using bacterial, fungal, plant, and mammalian cells (*E. coli*, yeast, microalgae, and human). AXT is one of the most potent antioxidants known in nature, and the label‐free high‐throughput screening for this type of molecule is of particular interest for biomanufacturing applications.^[^ ^29^ ^]^ Our system is capable of tracking the dynamic AXT accumulation process with high throughput (≈2700 events min^−1^). Thus, resonance band‐based monitoring of AXT production is greatly improved using the pDEP‐DLD‐RFC and can be employed in efforts to elevate overall system productivity (e.g., monitoring the microalgal cultivation to synchronize cells to a high‐AXT state).

Nonresonance band‐based profiling was also demonstrated using the pDEP‐DLD‐RFC chip to track TAG content. TAG is a potential source of biofuels and food/nutrients, thus such tools for the label‐free, noninvasive characterization cellular TAG factories are highly valuable.^[^ ^16^, ^30^ ^]^ Importantly, the Raman intensity for TAG is relatively high among other nonresonance peaks in a fs‐SCRS, thus shorter acquisition time (millisecond level, 200 ms used herein) is sufficient. However, longer acquisition times are frequently required to obtain high‐quality signals for weaker nonresonance peaks. CD band‐based profiling of metabolic activity was successfully used to model such applications. Indeed, pDEP‐DLD‐RFC enabled nonresonance band based, simultaneous profiling of multiple pivotal metabolic phenotypes, such as TAG content, DU values, metabolic vitality, AST, and metabolic state (which can discriminate cancer types via metabolic heterogeneity and metabolite‐conversion networks).

The rich information content within such fs‐SCRS suggests that there are numerous additional applications for pDEP‐DLD‐RFC, such as simultaneous quantification of intracellular energy‐storage (e.g., proteins, lipids, and starch) compounds,^[^ ^4d^ ^]^ tracking of substrate assimilation via stable isotope (i.e., ^13^C and ^15^N) labeling,^[^ ^1^ ^]^ interrogation of microbiota metabolic heterogeneity,^[^ ^31^ ^]^ antimicrobial or anticancer drugs screening, and quality assessment of live‐cell therapeutics (e.g., CAR‐T).^[^ ^32^ ^]^ Moreover, the emergence of reference fs‐SCRS databases for specific organisms suggests the potential use of pDEP‐DLD‐RFC for direct profiling of microbial composition in a human, environmental, or industrial microbiota.^[^ ^7^, ^24^ ^]^

In summary, mass‐spectrometry and fluorescence‐based flow cytometry systems have transformed life science and medical research/practice over the last few decades. However, the need to label (for fluorescence) or destroy (for mass‐spec) the target cell has hindered their even wider deployment.^[^ ^33^ ^]^ The introduction of this pDEP‐DLD‐RFC system provides a new tool to tackle this challenge. Its label‐free, noninvasive, information‐rich, universally applicable, and high‐throughput nature suggests Raman‐based flow cytometry can start to serve a diverse array of novel applications that involve metabolic phenome profiling.

## 4 Experimental Section

### Design and Fabrication of the pDEP‐DLD‐RFC Chip

The pDEP‐DLD‐RFC chip was fabricated using quartz by bonding the bottom ITO‐electrode array layer and a top thin cover slice (200 µm in thickness) with double‐sided tape (Figure 1C; and Figure S1, Supporting Information). The bottom ITO‐electrodes array layer was designed using AutoCAD 2022 (Autodesk, US). The 60 electrode pairs are 110 nm in height and 30 µm in width and are distributed with a 50 µm spacing. The channel for loading cells and Raman acquisition is 1 mm in width and 50 µm in height. The quartz (coated with a 110 nm thick ITO layer) was spin‐coated with a 2 µm thick positive photoresist layer (RZJ‐306, Suzhou Ruihong, CN), exposed to UV light and developed to fabricate the bottom ITO‐electrode array layer. The developed substrate was etched in an ITO etchant (FeCl_3_ : HCl = 1 : 2) and cleaned with acetone.

### System Setup

As shown in Figure 1B, cells were loaded via PEEK tubing with a small inner diameter (≈305 µm; Cole‐Parmer, US), which was connected to the microfluidic device and a syringe equipped on the pumps (LSP01‐2A, Longer Pumps, CN). An alternating current (ac) of 18 volts peak to peak (Vp‐p) at 1 MHz (for yeast, *E. coli*, and cancer cells) and 400 KHz (for *H. pluvialis* cells) frequencies were generated by an arbitrary function generator (DG4620, RIGOL Ltd., CN). A continuous sinusoid wave was outputted to trap the cells for fs‐SCRS acquisition. A normally open relay was connected between the function generator and the electrodes on the microfluidic chip and activated by a digital input/output (I/O) unit (DIO‐1616LX‐USB, CONTEC Ltd., US). This system triggered immediate pDEP interruptions for the on‐demand release of cells, which enabled a dielectrophoretic cell trapping and focusing efficiency of >90% (Movies S1 and S2, Supporting Information).

Raman microscopy was conducted using a FlowRACS instrument (Qingdao Single‐cell Biotech, CN). The FlowRACS instrument was utilized a Nd:YAG 532 nm laser emitter as the excitation light source (300 mW was used in all the experiments), with a 60× water objective (NA = 1.0, Olympus, JP) to focus the laser beam on the sample. An electron‐multiplying charge‐coupled device (EMCCD; Newton DU970N‐BV, Andor, UK) was used for collecting SCRS. A 1200 lines mm^−1^ grating was used for the measurements.

QSpec software was used (Qingdao Single‐cell Biotech, CN) to control the electronics (EMCCD, function generator, relay, etc.) and adjust the system parameters (e.g., acquisition time and trap and release frequency). All the pDEP‐DLD‐RFC units, including the microfluidic device, Raman system, and function generator were integrated with QSpec, which was operated in automatic mode.

During the pDEP‐DLD‐RFC, the beads/cell associated Raman spectra based upon the SNR, which was defined as the ratio between the target peak intensity and the standard deviation (SD) of the peaks between 1800 and 1840 cm^−1^, were filtered. The target peaks were present at 1001 cm^−1^ for PS beads, 2950 cm^−1^ for PMMA beads, 1516 cm^−1^ for AXT‐producing *H. pluvialis* cells, and 2900 cm^−1^ for TAG‐producing and D_2_O‐labeled yeast, *E. coli* and cancer cells. The background Raman signal was determined via an empty pDEP‐DLD‐RFC run. With a 2 ms acquisition time, all background SNR was <5, hence a SNR of ≥5 was used for obtaining Raman spectra of PS beads, PMMA beads, and AXT‐producing *H. pluvialis* cells. With a 200 ms acquisition time, all background SNR was <50, hence a SNR of ≥50 was used for obtaining Raman spectra for TAG‐producing yeast. With a 2 s acquisition time, all background SNR was <150 for D_2_O‐labeled yeast and *E. coli* cells, and <180 for cancer cells, hence a SNR of ≥150 was used for obtaining D_2_O‐labbeled yeast and *E. coli* cells Raman spectra, and ≥180 for cancer cells. All the data generated through these means were processed using R (version 4.1.1).

### Evaluation of pDEP‐DLD‐RFC Performance with Beads

Uniform 20 µm diameter PS and PMMA beads were employed to evaluate the pDEP‐DLD‐RFC performance. The beads were loaded into the chip at high concentration (>10^6^ beads mL^−1^) and their Raman spectra acquired under a trap‐free condition (as the beads are not sensitive to the pDEP force). The acquisition time was set as 2 ms for collecting each raw Raman spectrum for the beads. The intensities at 1001.1 cm^−1^ (one of the most prominent peaks for PS beads; defined as *I*_1001_‐ *I*_1800_) and 2950 cm^−1^ (one of the most prominent peaks for PMMA beads; defined as *I*_2950_‐ *I*_1800_) were extracted from the raw Raman spectra (*n* > 8000), which enabled the standard deviation and discrimination accuracy to be derived.

### Preparation of *H. pluvialis*

*H. pluvialis* (SAG‐34) cells were cultured as previously described.^[^ ^4a^ ^]^ Briefly, a single colony from basal medium plates^[^ ^34^ ^]^ was inoculated into liquid basal medium at 22 °C under continuous low light illumination (20 µmol photons m^−2^ s^−1^) with manual shaking once per day. To profile the AXT productivity, exponentially growing cells were resuspended into modified BBM medium (without added nitrogen and containing 10 mm sodium acetate) in triplicate and exposed to continuous 150 µmol photons m^−2^ s^−1^ illumination. A series of pDEP‐DLD‐RFC measurements were performed from 0 to 4 days. To compare the effect of nitrogen deficiency and high light, exponentially growing cells were cultured in basal medium under high light (150 µmol photons m^−2^ s^−1^) or in modified BBM medium under low light (20 µmol photons m^−2^ s^−1^) for 3 days, then underwent pDEP‐DLD‐RFC. All the harvested cells were filtered with a cell strainer (40 µm microporous membrane filtration) to remove debris and cell clusters, centrifuged at 2000 g and washed in sterile deionized water three times and resuspended in 1% Pluronic F‐127 (Sigma‐Aldrich, US) to prevent cell adhesion to electrodes (especially at the laser spot). According to the loading velocity of 40 µL min^−1^ and the ≈2700 events min^−1^ throughput, the ideal cell concentration for loading is ≈6.75 × 10^4^ cells mL^−1^ (≈2700 cells in 40 µL loading buffer).

### Preparation of TAG‐Producing Yeast

The “NoDGAT2A” TAG‐producing yeast strain was derived by transforming the *S. cerevisiae* strain H1246 (which is unable to synthesize TAG due to *DGA1*, *LRO1*, *ARE1*, and *ARE2* knockouts) with a vector that harbors the *NoDGAT2A* gene (encoding *Nannochloropsis oceanica* DGAT that assembles TAG).^[^ ^16^ ^]^ The cells were cultured and prepared as previously reported.^[^ ^16^ ^]^ Briefly, the strains were maintained at 30 °C and stored at 4 °C on 2% agar plates with selective culture medium (SCM; 0.67% yeast nitrogen base, 2% glucose, and 0.074% ‐His/‐Ura DO supplement) solidified. The cells were activated by inoculating a single colony into 10 mL liquid SCM and cultured in an orbital shaker at 30 °C and 200 rpm for 12 h. To induce TAG production, 200 µL cell suspensions (harvested by centrifugation after being cultured in SCM) were cultured in the induction medium (IM; 0.67% yeast nitrogen base, 0.074% ‐His/‐Ura DO supplement, 1% raffinose, and 2% galactose). The cells were centrifuged at 2000 g and washed three times with sterile deionized water, collected and resuspended in 1% Pluronic F‐127 (Sigma‐Aldrich, US) for pDEP‐DLD‐RFC. According to the loading velocity of 40 µL min^−1^ and the ≈270 events min^−1^ throughput, the ideal cell concentration for loading is ≈6.75 × 10^3^ cells mL^−1^ (≈270 cells in 40 µL loading buffer).

### Preparation of D_2_O‐Fed Yeast

*S. cerevisiae* strain BY‐4742 was maintained at 30 °C and stored at 4 °C on 2% agar YPD medium plates. The cells were activated by inoculating a single colony into 10 mL liquid YPD medium and cultured in an orbital shaker at 30 °C and 200 rpm for 12 h. To compare the metabolic activity of cells at various time points, cells cultured for 12 or 36 h were harvested and incubated for 4 h with 40% D_2_O in YPD medium (with a 10 mL working volume). The cells were centrifuged at 2000 g and washed three times with sterile deionized water, collected and resuspended in 1% Pluronic F‐127 (Sigma‐Aldrich, US) for pDEP‐DLD‐RFC. According to the loading velocity of 40 µL min^−1^ and the ≈30 events min^−1^ throughput, the ideal cell concentration for loading is ≈7.50 × 10^2^ cells mL^−1^ (≈30 cells in 40 µL loading buffer).

### Preparation of TAG‐Producing D_2_O‐Fed Yeast

The NoDGAT2A cells were activated by inoculating a single colony into 10 mL liquid SCM and cultured in an orbital shaker at 30 °C and 200 rpm for 12 h. The cells (harvested by centrifugation after being cultured in SCM) were cultured in induction medium to induce TAG production. Cells were harvested at each time point (day 0, day 0.5/12 h, day 1, day 2, and day 4), and incubated for 4 h with 40% D_2_O in induction medium (with a 10 mL working volume). According to the loading velocity of 40 µL min^−1^ and the ≈30 events min^−1^ throughput, the ideal cell concentration for loading is ≈7.50 × 10^2^ cells mL^−1^ (≈30 cells in 40 µL loading buffer).

### Preparation of Cancer Cells

Human bladder (T24), lung (A549), renal (OSRC‐2), and breast (MCF‐7) cancer cells were cultured using Dulbecco's modified Eagle's medium (DMEM; Thermo Fisher, US) supplemented with 1% penicillin/streptomycin (10 000 units mL^−1^) and 10% fetal bovine serum (Thermo Fisher, US) in a 100 mm tissue culture dish (Thermo Fisher, US) in a humidified 37 °C incubator with 5% CO_2_. When nearly confluent, the cells were detached from the dish at 37 °C using 2 mL 0.25% w/v trypsin‐EDTA (Gibco) for 2–3 min, the cells were centrifuged at 300 g for 5 min, washed three times with PBS buffer and resuspended in an isotonic low‐conductivity DEP buffer (8.6% sucrose and 0.3% dextrose). According to the loading velocity of 40 µL min^−1^ and the ≈30 events min^−1^ throughput, the ideal cell concentration for loading is ≈7.50 × 10^2^ cells mL^−1^ (≈30 cells in 40 µL loading buffer).

### 4.1 *E. Coli* Treatment

ATCC‐25922 *E. coli* were purchased from the American Type Culture Collection (ATCC; US). *E. coli* 1 768 859 is a clinical isolate identified by 16S recombinant DNA sequencing that was provided by Zhujiang Hospital (CN). Both strains were cultured in Cation‐Adjusted Mueller‐Hinton Broth (CAMHB; Oxoid, UK). Notably, there are differences in D_2_O intake rate between susceptible and resistant strains that are more profound when adding antimicrobials ahead of D_2_O, than when adding them simultaneously.^[^ ^24^, ^35^ ^]^ Therefore, in order to maximize such differences when distinguishing susceptible and resistant strains, the cells were incubated with drugs for 1 h, and then incubated with 50% D_2_O for 2 h for AST assays. After incubation, the cells were harvested and washed three times with sterile deionized water centrifuged at 3000 g and resuspended in 1% Pluronic F‐127 (Sigma‐Aldrich, US). According to the loading velocity of 40 µL min^−1^ and the ≈30 events min^−1^ throughput, the ideal cell concentration for loading is ≈7.50 × 10^2^ cells mL^−1^ (≈30 cells in 40 µL loading buffer).

### Deep Learning with CNNs

The ramanomes acquired by pDEP‐DLD‐RFC were analyzed using a CNN structure to classify different cell types. The CNN structure had six initial convolutional layers, six residual layers, six Max‐pooling layers, and one fully connected layer. The training process was performed on four NVIDIA GTX‐3080 GPUs, CUDA 11.7, and cuDNN 8.0.3 environments. For the training process, half the amount of SCRS were randomly picked as the training dataset and half the amount of SCRS were used as the test dataset for each cell type. Feature extraction was performed for each SCRS by the convolutional of layers in a 1D CNN, batch normalization layers were used for internal data standardization. Max‐pooling layers were employed to compress the features, which were inputted into a fully connected layer to complete classification. The network output was converted into a probability value to provide a classification result. The CNN was trained for 200 epochs using cross‐entropy as the loss function, mini‐batch gradient descent with batch size of 32, an initial learning rate of 0.0001, and a slightly regularizing weight decay of 0.000 001 as the optimizer.

### Visualizing pDEP‐DLD‐RFC Derived Ramanomes with t‐SNE Plots

A *t*‐SNE analysis was used to better understand the features highlighted by the CNN, which was implemented in Scikit‐learn^[^ ^36^ ^]^ to visualize the activations in the final Max‐Pooling layer. Unsupervised learning was used, i.e., without cell type labels for the 64 features in the CNN model. Distinct clusters were identified in the 2D *t*‐SNE plots, suggesting that the CNN model effectively obtained important Raman spectra information and omitted artificial noise. The implementation code of the proposed method was programmed by Pytorch 1.18.0 and Scikit‐learn 0.24.2 based on the Python 3.7 environment.

### Intra‐Ramanome Correlation Analysis (IRCA)

The SCRS were normalized, followed by the IRCA using a previously reported customized computational pipeline for data analysis and result visualization.^[^ ^8^ ^]^ SCRS from different batches of ramanome profiling experiments may have distinct spectral ranges and resolution due to technical variation (e.g., change of data collector and batch). Therefore, prior to computing the IRCA networks, all the ramanomes were standardized in three steps: i) for spectral range, only the “fingerprint area” (600–1800 cm^−1^) was extracted; ii) spectral resolution was simulated to 1 cm^−1^, via the interpolation algorithm; iii) spectral normalization was performed via division by its area.

For each IRCN, the correlation matrix of each ramanome was constructed by calculating the Pearson correlation coefficient (rho/*ρ*) for all possible pairwise Raman peak combinations among all the sampled cells. The Raman peak pairs with a significant correlation (*p* <0.05) were considered candidates that potentially indicate links between two metabolites, while those with strong negative correlations suggested potential conversions among two metabolites (*ρ* ≤−0.6, *P* <0.05). The *igraph*‐1.2.8 package in R (version 4.1.1) was used to derive key network properties and visualize the IRCNs (from either specific subset of or all the Raman peaks). For global IRCNs, all Raman peaks were used. To facilitate visualization, simplified IRCNs were also derived, which contain only characteristic/marker peaks.

### Statistical Analysis

All the raw SCRSs were first preprocessed with in‐house scripts RamEx, including background subtraction and baseline correction with a polynomial algorithm (seventh degree). Data presentation (e.g., mean ± SD), sample size, statistical tests, and software (Python and R) were specified when applicable in Results, Methods, and Figure Legends.

## Conflict of Interest

J.X. and B.M. are on the scientific board of Qingdao Single‐cell Biotechnology Co., Ltd. The authors declare that they have no other competing interests.

## Author Contributions

X.W., L.R., Z.D., and Y.H. contributed equally to this work. X.W., L.R., Z.D., and Y.H. designed and carried out the experiments. X.W. and Z.D. designed the microfluidic devices and set up the pDEP‐DLD‐RFC platform. Y.L. set up and optimized the FlowRACS instrument. L.R. designed QSpec. J.Z. and M.L. prepared the cell samples. Y.H., L.S., R.C., and T.J. analyzed SCRS. X.W., J.X. and B.M. wrote the manuscript. J.X. and B.M. conceived the project.

## Supporting information

Supporting information

Supplemental Movie 1

Supplemental Movie 2

## Data Availability

The data that support the findings of this study are available from the corresponding author upon reasonable request.
