# Supplementary material for: Robust Spontaneous Raman Flow Cytometry for Single‐Cell Metabolic Phenome Profiling via pDEP‐DLD‐RFC
Source: Adv Sci (Weinh). 2023 Mar 4;10(16):2207497. doi: 10.1002/advs.202207497 (PMC10238217; doi:10.1002/advs.202207497)
Supplement: Supplementary file 1 — Supporting information [file ADVS-10-2207497-s001.pdf]

ADVANCED SCIENCE
Open Access

Supporting Information

for *Adv. Sci.*, DOI 10.1002/advs.202207497

Robust Spontaneous Raman Flow Cytometry for Single-Cell Metabolic Phenome Profiling via pDEP-DLD-RFC

Xixian Wang, Lihui Ren, Zhidian Diao, Yuehui He, Jiaping Zhang, Min Liu, Yuandong Li, Lijun Sun, Rongze Chen, Yuetong Ji, Jian Xu and Bo Ma**

Supporting Information

Robust spontaneous Raman flow cytometry for single-cell metabolic phenome profiling via pDEP-DLD-RFC

Xixian Wang, Lihui Ren, Zhidian Diao, Yuehui He, Jiaping Zhang, Min Liu, Yuandong Li, Lijun Sun, Rongze Chen, Yuetong Ji, Jian Xu, Bo Ma**

Figure S1. pDEP-DLD-RFC chip fabrication.
Figure S2. pDEP-DLD-RFC system operation.
Figure S3. Global IRCNs for the *NoDGAT2A*-expressing yeasts sampled at various time points from within the SCM or IM.
Figure S4. AXT content profiling for *H. pluvialis* under high light plus nitrogen depletion.
Figure S5. AXT content profiling for *H. pluvialis* under either high light or nitrogen depletion.
Figure S6. Ramanome-based profiling of TAG productivity in *NoDGAT2A*-expressing yeast.
Figure S7. Ramanome-based degree of lipid unsaturation (DU) profiling in *NoDGAT2A*-expressing yeast.
Figure S8. Ramanome-based CD Ratio (CDR) profiling in yeast.
Figure S9. Global IRCNs for *NoDGAT2A*-expressing yeast that were supplemented with D_2O following various temporal incubations in induction medium.
Figure S10. Global IRCNs for four cancer cell lines: T24 (bladder), A549 (lung), OSRC-2 (renal) and MCF-7 (breast).
Movie S1. pDEP-DLD based cell focusing.
Movie S2. Single-cell trapping and release after pDEP-DLD based cell focusing.

Supplementary Figures

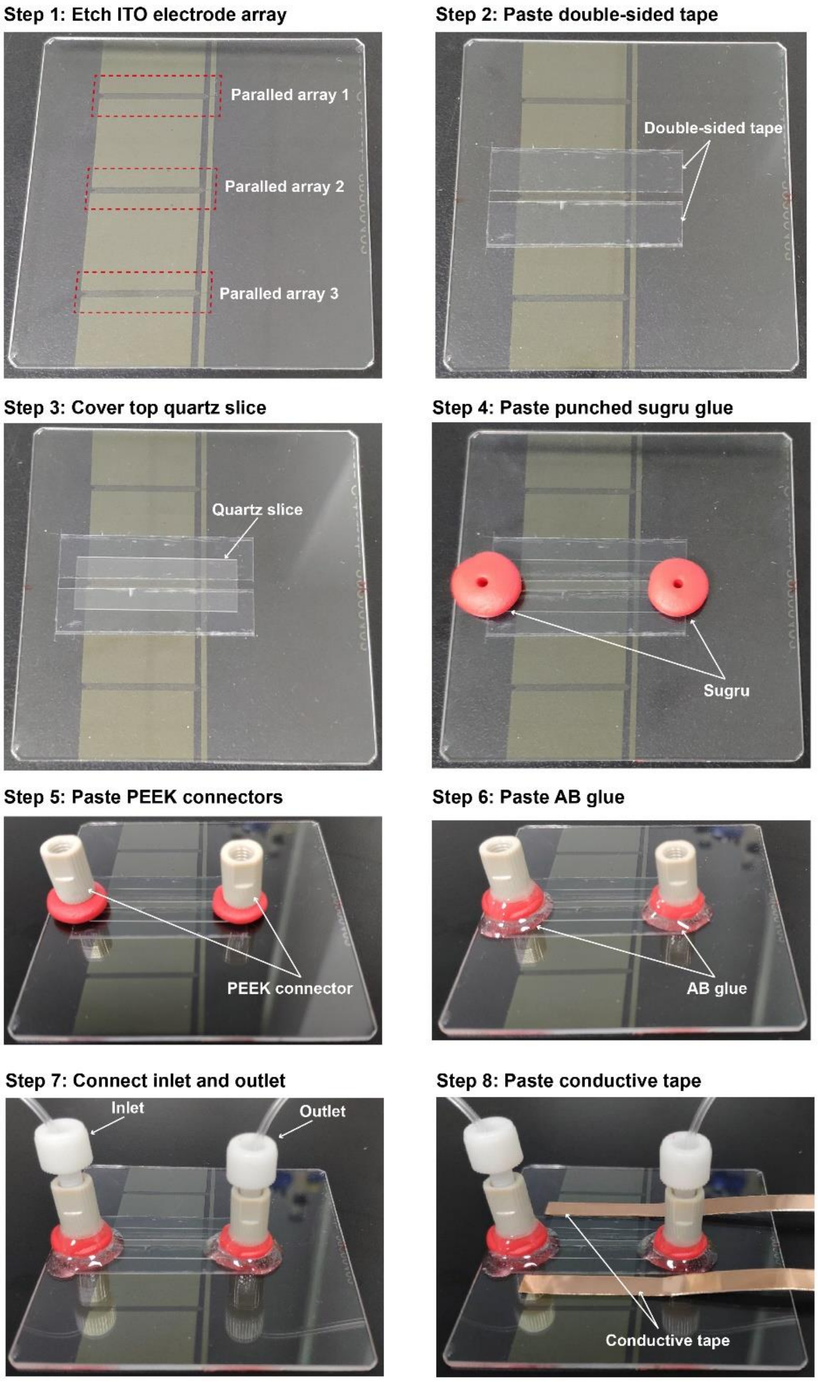

Figure S1. pDEP-DLD-RFC chip fabrication. Step 1: etching the ITO electrode array; **Step 2:** pasting the double-sided tape to construct the loading channel; **Step 3:** covering the top quartz slice to facilitate SCRS acquisition; **Step 4:** applying the punched surgru glue to connect the PEEK connector; **Step 5:** placing the PEEK connector on surgru glue; **Step 6:** applying the AB glue to immobilize the PEEK connector; **Step 7:** connecting the inlet and outlet for loading and exporting cells; **Step 8:** conductive tape placement for the pDEP.

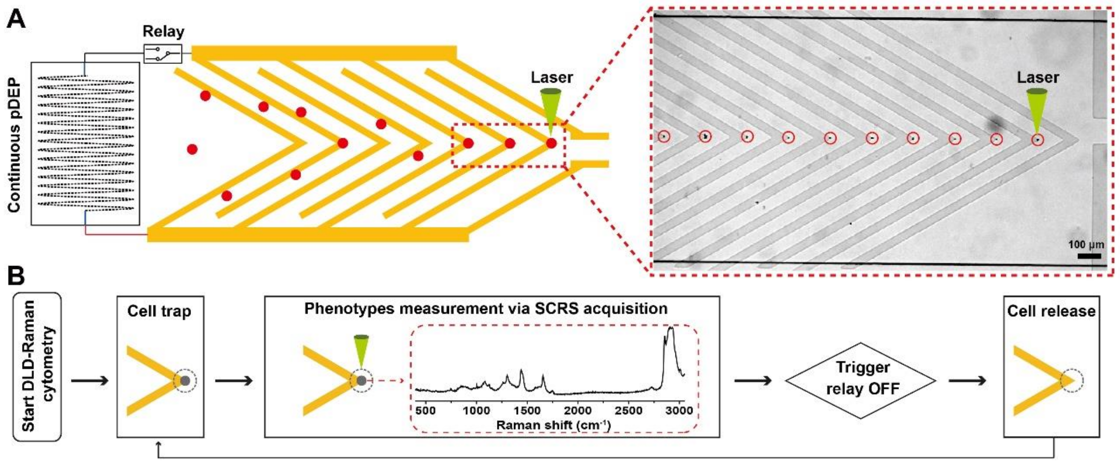

Figure S2. pDEP-DLD-RFC system operation. (A) Operation strategy to enable high throughput SCRS profiling. Fast-moving single cells are focused by pDEP-DLD and delivered to the Raman laser by repeated trap-releases. An inset bright-field image displays single cells that are trapped and released (a still extracted from Movie S2). **(B)** QSpec software workflow for efficient SCRS acquisition. Scale bar, 100 μm.

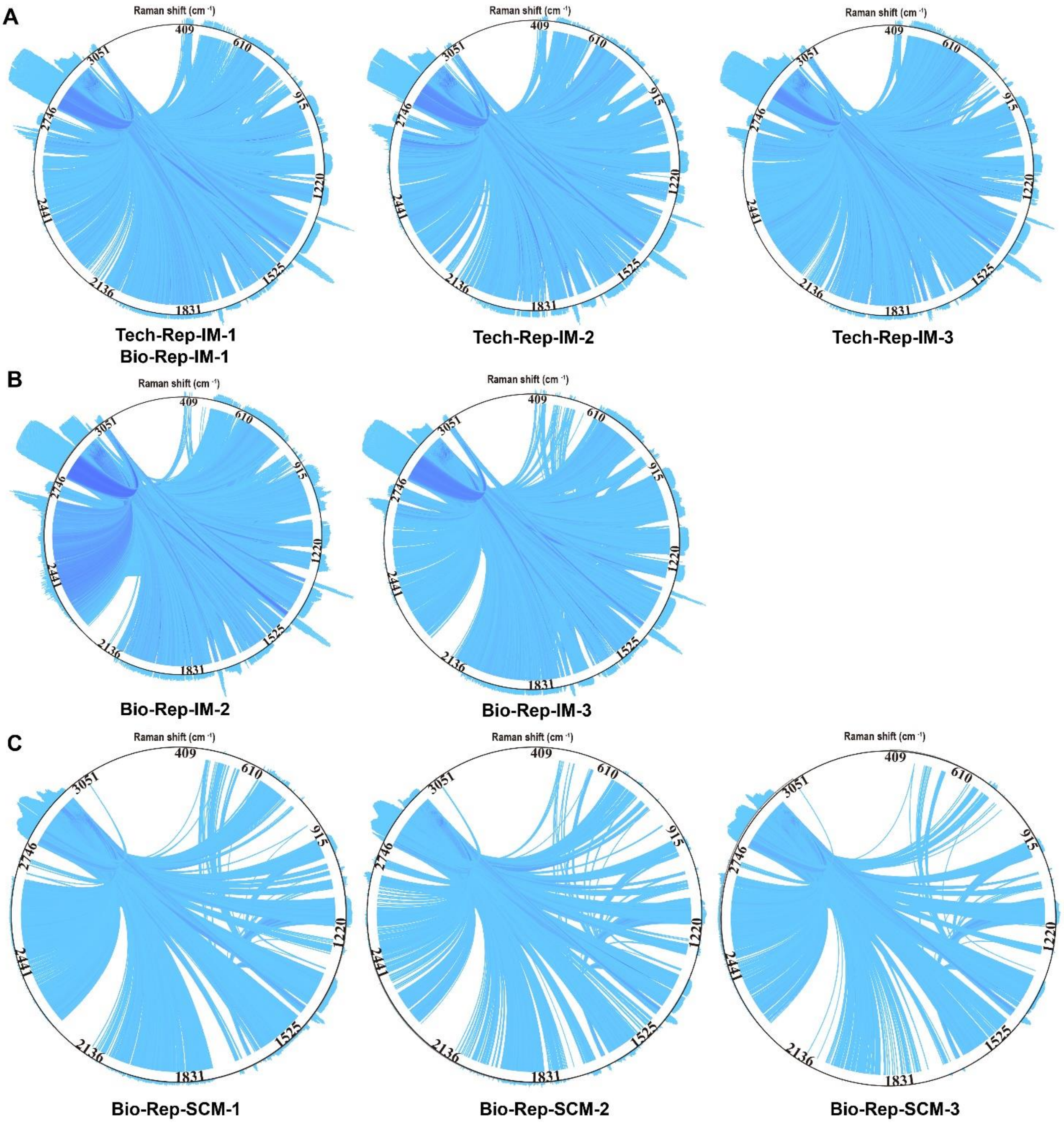

Figure S3. Global IRCNs for the *NoDGAT2A*-expressing yeasts sampled at various time points from within the SCM or IM. IM IRCNs for the **(A)** technical replicates, **(B)** biological replicates. **(C)** IRCNs for the Biological replicates in SCM.

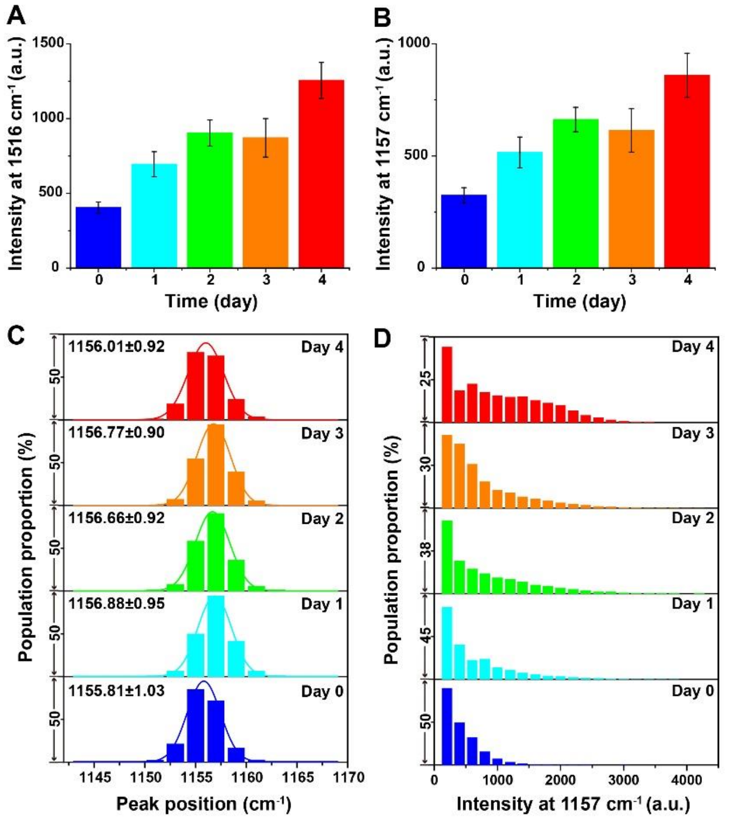

Figure S4. AXT content profiling for *H. pluvialis* under high light plus nitrogen depletion. Average Raman intensity at **(A)** 1516 cm^{-1} and **(B)** 1157 cm^{-1}. Error bars: SD of three replicates. **(C)** Distribution around the 1157 cm^{-1} peak during the four-day cultivation. **(D)** Temporal tracking of AXT-production by *H. pluvialis* cells during the four-day cultivation. Error bars indicate the SD of three independent experiments.

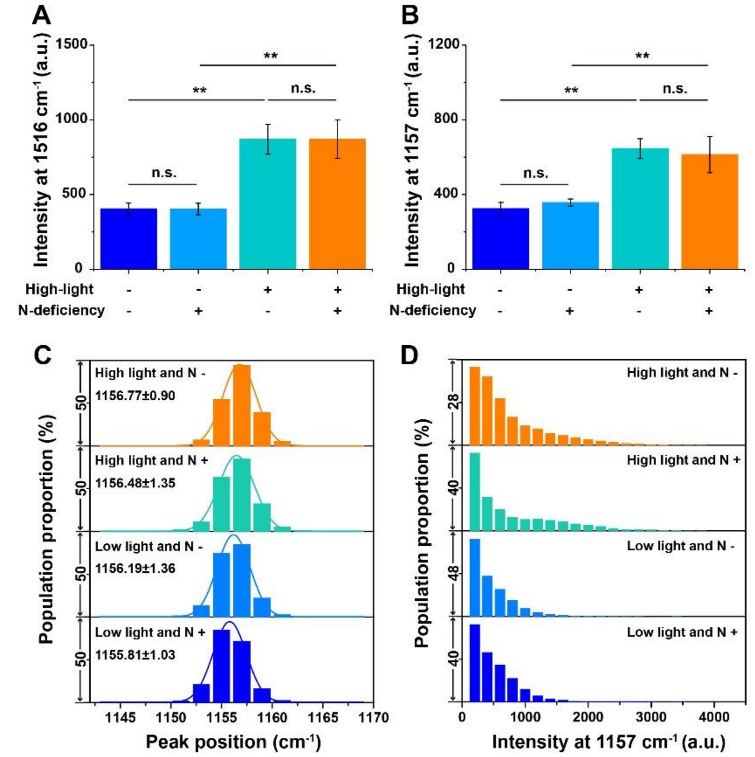

Figure S5. AXT content profiling for *H. pluvialis* under either high light or nitrogen depletion. Average Raman intensity at **(A)** 1516 cm^{-1} and **(B)** 1157 cm^{-1} under different stress conditions. Error bars: SD of three replicates. $^{**}P < 0.01$; n.s., not significant. **(C)** Distribution around the 1157 cm^{-1} peak under different stress conditions. **(D)** Comparison of AXT-production by *H. pluvialis* cells with various stress conditions. Error bars indicate the SD of three independent experiments. The statistic difference in (**A**) and (**B**) was analyzed using the unpaired two-tailed Student's *t*-test.

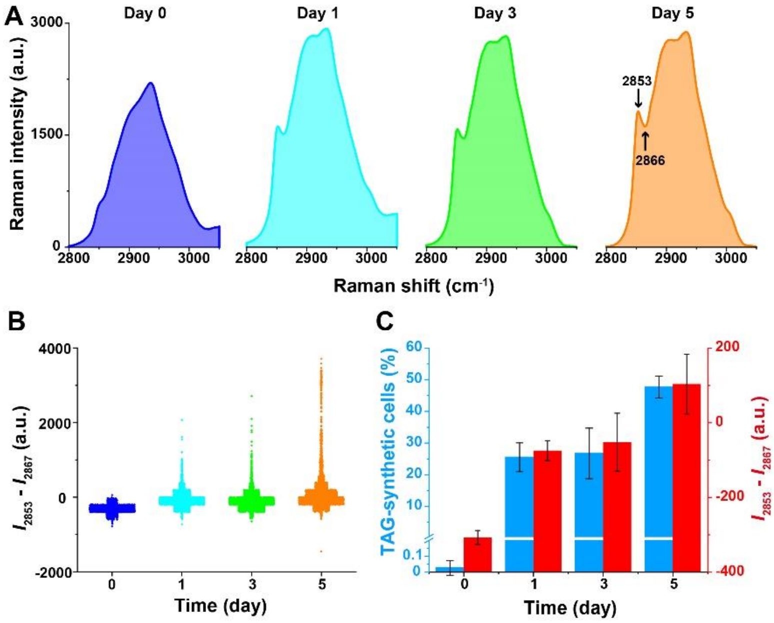

Figure S6. Ramanome-based profiling of TAG productivity in *NoDGAT2A*-expressing yeast. (A) The average SCRS at 2800 to 3050 cm^{-1} (n >3500) for *NoDGAT2A*-expressing yeast cells, showing a gradual TAG production increase over the induction time. **(B)** Distribution of "I_{2853} - I_{2866}" (representing TAG content). **(C)** Percentage of TAG-synthetic cells (blue/left, showing those I_{2853} - I_{2866} > 0) and average "I_{2853} - I_{2866}" (red/right). Error bars indicate the SD of three independent experiments.

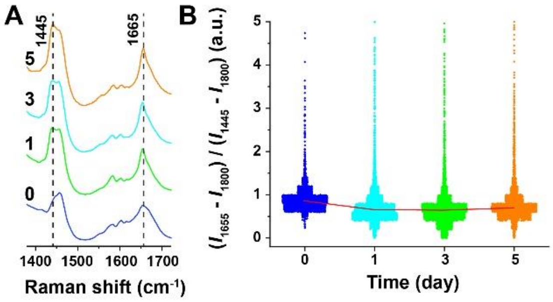

Figure S7. Ramanome-based degree of lipid unsaturation (DU) profiling in *NoDGAT2A*-expressing yeast. (A) The average SCRS at 1400 to 1700 cm^{-1} (n >3500) for *NoDGAT2A*-expressing yeast cells. **(B)** Distribution around "(I_{1665} - I_{1800}) / (I_{1445} - I_{1800})" (representing DU).

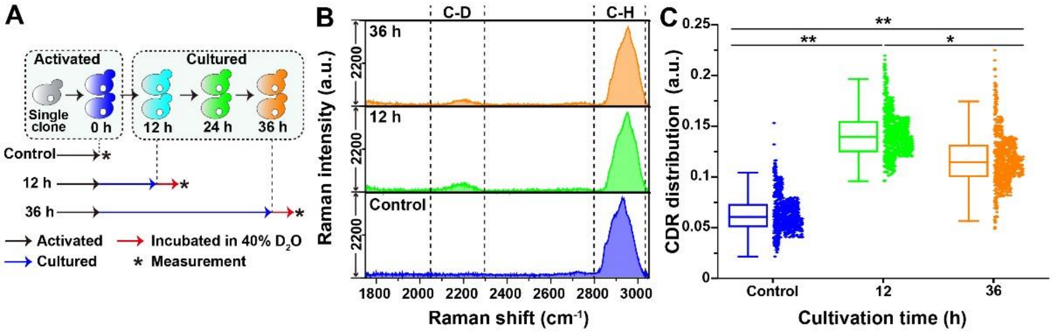

Figure S8. Ramanome-based CD Ratio (CDR) profiling in yeast. (A) Yeast preparation with different culture durations and incubated with D_2O under identical conditions. **(B)** The average SCRS at 1800 to 3000 cm^{-1} (n >3500) incubated with D_2O. **(C)** CDR distribution (representing metabolic activity). The lines in the box indicate the mean value. *P <0.05; **P <0.01. The statistic difference in (C) was analyzed using the unpaired two-tailed Student t-test.

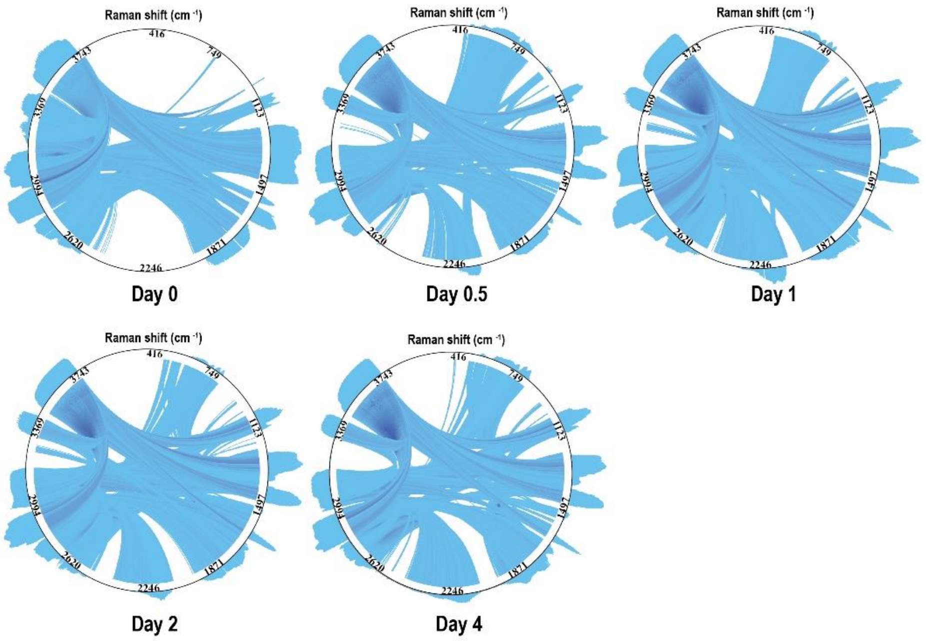

Figure S9. Global IRCNs for *NoDGAT2A*-expressing yeast that were supplemented with D_2O following various temporal incubations in induction medium. IRCNs derived from the yeast ramanomes.

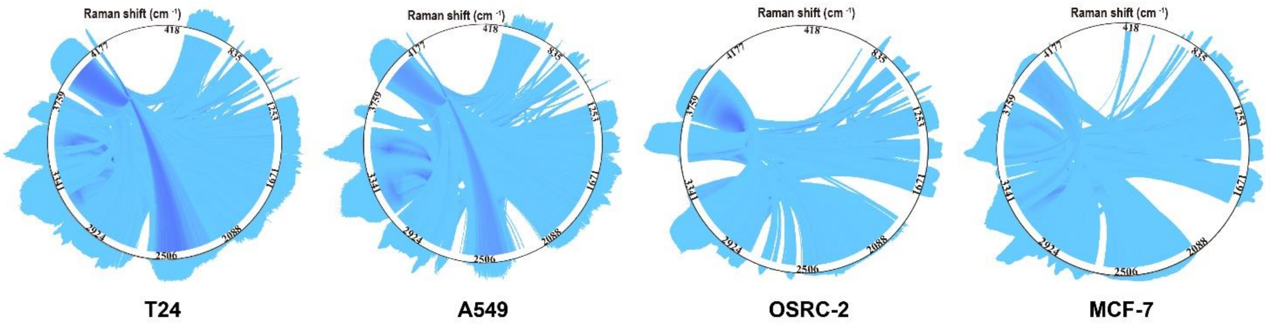

Figure S10. Global IRCNs for four cancer cell lines: T24 (bladder), A549 (lung), OSRC-2 (renal) and MCF-7 (breast). IRCNs derived from cancer ramanomes.
